# Expert consensus on the identification, diagnosis, and treatment of neurotrophic keratopathy

**DOI:** 10.1186/s12886-021-02092-1

**Published:** 2021-09-08

**Authors:** Reza Dana, Marjan Farid, Preeya K. Gupta, Pedram Hamrah, Paul Karpecki, Cathleen M. McCabe, Lisa Nijm, Jay S. Pepose, Stephen Pflugfelder, Christopher J. Rapuano, Arvind Saini, Sarah N. Gibbs, Michael S. Broder

**Affiliations:** 1 Massachusetts Eye and Ear, Harvard Medical School Department of Ophthalmology, Boston, MA 02114 USA; 2 University of California, Irvine School of Medicine, 850 Health Sciences Rd, Irvine, CA 92697 USA; 3 Duke University Eye Center, 4709 Creekstone Drive, Suite 100, Durham, NC 27703 USA; 4 Tufts Medical Center, Tufts University School of Medicine, 800 Washington St, Boston, MA 02111 USA; 5 UPike College of Optometry/Kentucky Eye Institute, 147 Sycamore Street, Pikeville, KY 41501 USA; 6 The Eye Associates, 2111 Bee Ridge Rd, Sarasota, FL 34239 USA; 7 Warrenville EyeCare and LASIK, 2S631 Illinois Route 59, Suite A, Warrenville, IL 60555 USA; 8 University of Illinois Eye and Ear Infirmary, 1855 W Taylor St, Chicago, IL 60612 USA; 9 Pepose Vision Institute, 1815 Clarkson Rd, Chesterfield, MO 63017 USA; 10 Washington University School of Medicine, Department of Ophthalmology and Visual Science, 660 Euclid Avenue, St. Louis, MO 63110 USA; 11 Cullen Eye Institute, Baylor College of Medicine, 6565 Fannin St, NC-505, Houston, TX 77030 USA; 12 Wills Eye Hospital, 840 Walnut St, Philadelphia, PA 19107 USA; 13 Integrity Eye, 1955 Citracado Parkway, Escondido, CA 92029 USA; 14 Partnership for Health Analytic Research (PHAR), LLC, 280 S Beverly Dr Suite 404, Beverly Hills, CA 90212 USA

**Keywords:** Corneal diseases, Keratitis, Corneal epithelium, Trigeminal nerve diseases, Consensus

## Abstract

**Background:**

Neurotrophic keratopathy (NK) is a relatively uncommon, underdiagnosed degenerative corneal disease that is caused by damage to the ophthalmic branch of the trigeminal nerve by conditions such as herpes simplex or zoster keratitis, intracranial space-occupying lesions, diabetes, or neurosurgical procedures. Over time, epithelial breakdown, corneal ulceration, corneal melting (thinning), perforation, and loss of vision may occur. The best opportunity to reverse ocular surface damage is in the earliest stage of NK. However, patients typically experience few symptoms and diagnosis is often delayed. Increased awareness of the causes of NK, consensus on when and how to screen for NK, and recommendations for how to treat NK are needed.

**Methods:**

An 11-member expert panel used a validated methodology (a RAND/UCLA modified Delphi panel) to develop consensus on when to screen for and how best to diagnose and treat NK. Clinicians reviewed literature on the diagnosis and management of NK then rated a detailed set of 735 scenarios. In 646 scenarios, panelists rated whether a test of corneal sensitivity was warranted; in 20 scenarios, they considered the adequacy of specific tests and examinations to diagnose and stage NK; and in 69 scenarios, they rated the appropriateness of treatments for NK. Panelist ratings were used to develop clinical recommendations.

**Results:**

There was agreement on 94% of scenarios. Based on this consensus, we present distinct circumstances when we strongly recommend or may consider a test for corneal sensitivity. We also present recommendations on the diagnostic tests to be performed in patients in whom NK is suspected and treatment options for NK.

**Conclusions:**

These expert recommendations should be validated with clinical data. The recommendations represent the consensus of experts, are informed by published literature and experience, and may improve outcomes by helping improve diagnosis and treatment of patients with NK.

## Background

Neurotrophic keratopathy (NK) is a rare degenerative corneal disease affecting approximately 5 per 10,000 people [1, 2]. Damage to the ophthalmic branch of the trigeminal nerve by conditions such as herpes simplex or zoster keratitis, intracranial space-occupying lesions, diabetes, or neurosurgical procedures [1] may result in decreased or absent corneal sensitivity. Damage to branches of the facial nerve may also affect the autonomic nervous system (sympathetic and parasympathetic), reducing tear production and secretion, and may lead to dry eye disease and NK [3]. Over time, epithelial breakdown, corneal ulceration, corneal melting (thinning), perforation, and loss of vision may occur.

Diagnosis is based on clinical history, eye examination, and testing to assess decreased corneal sensitivity and nerve damage [1]. Once diagnosed, NK can be classified into three stages using the Mackie classification, from the relatively mild Stage 1 (corneal epithelial changes) and moderate Stage 2 (corneal epithelial defect), to the more severe Stage 3 (corneal ulcer, perforation, melting) [4, 5]. Dua et al. [1] suggested a modification of the Mackie classification, incorporating corneal esthesiometry findings, which may be more clinically relevant by including specific reference to severity and prognosis. They define mild disease as epithelial changes only with no epithelial defect, manifestations of superficial punctate keratopathy and tear film instability, and a reduced or absent sensation in one or more quadrants of the cornea; moderate disease as epithelial defect with corneal anesthesia; and severe disease as stromal involvement, corneal ulcer or perforation, and corneal anesthesia. Management of NK is based on clinical severity, and treatment aims to stop the progression of corneal damage and promote epithelial healing [1]. Left untreated, NK can result in blindness [6]. However, as patients typically experience few symptoms in the earlier stages, diagnosis is often delayed.

While expert opinion on the diagnosis and treatment of NK has been published [1, 2, 7], no formal clinical guidelines exist. The modified Delphi panel method (or RAND/UCLA Appropriateness Method) has been used extensively to develop quality measures and clinical guidance in a variety of areas [8–12]. There is evidence that the resultant measures have content, construct, and predictive validity [9]. In addition, the method has been shown to produce guidance statements that improve health outcomes: patient care that was concordant with guidelines for coronary angiography, carotid endarterectomy, and coronary revascularization developed with the modified Delphi method produced better clinical outcomes than care that was discordant [10–13].

In this study, we conducted a RAND/UCLA modified Delphi panel to develop expert consensus on when to screen for and how best to diagnose and treat NK.

## Methods

We used the RAND/UCLA modified Delphi panel method [14–16]. This method is a formal group consensus process that systematically and quantitatively combines expert opinion and published literature by asking panelists to rate, discuss, and then re-rate various patient scenarios. Our panel included 11 experts (nine cornea specialists, and one comprehensive ophthalmologist and one optometrist both of whom underwent additional training in cornea and external disease) with an average of 21 years (range 10–34 years) of clinical experience and extensive experience treating patients with NK. Experts were from a variety of practice settings (six academic, four community/private practice, and one from both settings) and United States (US) regions (four South, three Northeast, two Midwest, and two West).

With two exceptions (RD, PH), the panel was double-masked while work was ongoing: the sponsor did not know the identity of the experts and experts did not know the identity of the sponsor until after the final manuscript of the work was completed. One expert (RD) served as the panel chair and was aware of the sponsor. A second expert (PH) inadvertently became aware of the sponsor during an unrelated conversation with the sponsor during the process. Experts received honoraria for their participation. The sponsor did not provide input on study design, methods, results, or interpretation of findings. The sponsor did not review or comment on the manuscript but was provided with a copy prior to submission. The panelists were unmasked after the manuscript was finalized but before submission in order to accurately disclose conflicts of interest. Modified Delphi panels do not involve human subjects as defined in 45 CFR part 46, thus institutional review board approval was not required.

Experts reviewed a summary of the relevant literature then rated 735 patient scenarios. Two reviews [1, 2], one meta-analysis [17], four randomized-controlled trials [18–21], and two comparative trials [22, 23] served as significant resources for the literature review. Overall, the quality of the evidence was low, although we did not conduct a formal assessment. Scenarios were developed collaboratively with experts via individual interviews. Experts provided input as to which clinical characteristics would lead them to suspect NK, which tests and examinations they may use to diagnose NK, and which treatments are available for NK. The scenarios were grouped into three sections and rated on a 1–9 scale. In Part I, comprising 646 scenarios, panelists rated whether a test of corneal sensitivity was warranted in specific clinical circumstances (where 1 = no, do not need to test corneal sensitivity and 9 = yes, absolutely need to test corneal sensitivity). Scenarios outlined various clinical circumstances, including whether a patient had epithelial changes/defect, a history of diabetes or corneal procedures, used contact lenses or eye drops, had reduced blink, dry eye, or vision changes. In Part II, comprising 20 scenarios, panelists considered the adequacy of specific tests and examinations to diagnose and stage NK (where 1 = inadequate and 9 = optimal). Scenarios were various combinations of qualitative/quantitative tests, tear or blink assessments, and imaging tests. In Part III, comprising 69 scenarios, panelists were asked to rate the appropriateness of various treatments (medical management, non-surgical interventions, and surgical interventions) for NK across three disease stages (where 1 = inappropriate, providing this treatment alone or in combination would likely be poor quality care; 5 = appropriate, providing this treatment alone or in combination may be effective, but is not optimal; and 9 = optimal, providing this treatment alone or in combination is the best quality of care available).

Experts rated these 735 scenarios twice: once before and once after an eight-hour virtual panel meeting. During the meeting, which took place in October 2020, experts were provided with a document showing their own rating, the group median, and the average distance from the median for each scenario. As is typical in the RAND/UCLA modified Delphi panel method, we defined disagreement as two or more ratings of 1–3 and two or more ratings of 7–9 [16]. Items without disagreement were grouped into three categories based on their median (1–3, 4–6, 7–9). During the professionally moderated group discussion, participants shared reasons for their ratings, particularly in areas of disagreement, but were not asked to reach consensus. Following this discussion, they completed the ratings a second time. These second-round ratings were analyzed using the method described for the first-round. Statements describing the consensus that emerged from the second-round ratings were developed and circulated to the experts, who commented and made changes for clarity.

## Results

The panel disagreed on only 6% of the 735 scenarios after the group discussion compared to 40% in the pre-meeting ratings (Table 1). After discussion, there was disagreement on 7% of the scenarios describing when it was appropriate to test for corneal sensitivity (Part I), on 10% of scenarios related to appropriate tests to diagnose NK (Part II), and on 3% of the treatment scenarios (Part III).

**Table 1:** Percent of scenarios with disagreement in first-round (pre-meeting) and second-round (post-meeting) ratings

	N	Round 1 disagreement	Round 2 disagreement
**Overall**	735	40%	6%
**Part I: Should a test of corneal sensitivity be performed?**	646	42%	7%
Scenarios that differed by degree of epithelial defect			
No epithelial changes (no staining) or defect	256	38%	1%
Newly observed epithelial changes (staining) but no defect	256	45%	14%
Newly observed defect	128	46%	3%
Scenarios that differed by history of diabetes			
No diabetes or with well-controlled diabetes without evidence of end-organ damage	320	32%	3%
Persistent poorly controlled diabetes and/or with evidence of end-organ damage	320	53%	10%
**Part II: Rate the adequacy tests to diagnose and stage neurotrophic keratopathy**	20	25%	10%
**Part III: Rate the appropriateness various treatments**	69	20%	3%
Scenarios that differed by disease stage			
Stage 1	23	26%	4%
Stage 2	23	17%	0%
Stage 3	23	17%	4%
Scenarios that differed by treatment type			
Medical management	27	22%	0%
Non-surgical intervention	24	25%	4%
Surgical intervention	15	13%	7%

### Corneal sensitivity testing

The panel acknowledged that clinical and non-clinical factors beyond those rated can change the index of suspicion for NK and may influence the decision to perform testing. Nonetheless, for 93% of these scenarios, the panel agreed on recommendations. These recommendations are grouped by whether the panel strongly recommended a test for corneal sensitivity (median 7–9) and when a test may be considered (median 4–6) (Table 2).

**Table 2:** Patient characteristics for when corneal sensitivity testing is strongly recommended or may be considered

Strongly recommended	May be considered
• Persistent epithelial defect that does not improve within 14 days • Painless, newly observed epithelial defect of unknown etiology • History of herpetic eye disease • History of procedures that might have damaged the trigeminal nerve or conditions that might have involved the trigeminal nerve • Pain in the affected eye and multiple, concurrent risk factors, such as persistent poorly controlled diabetes and either reduced blink or a history of corneal procedures	• Acquired limbal stem cell deficiency • Newly observed epithelial staining and persistent poorly controlled diabetes • Persistent poorly controlled diabetes and vision changes not ascribed to diabetic retinopathy or cataract (even in the absence of corneal findings)

The panel strongly recommended a test of corneal sensitivity in patients with a painless epithelial defect, or a persistent epithelial defect that does not improve within 14 days [24]. The panel also strongly recommended such a test in patients with a history of herpetic eye disease and in patients who have had procedures that might have damaged the trigeminal nerve (such as procedures for meningioma or schwannoma, radiofrequency ablation, or parotid gland surgery), or who have conditions that might have affected the trigeminal nerve (such as stroke, multiple sclerosis, or space-occupying intracranial masses). The index of suspicion for NK is lower for patients with pain in the affected eye. However, for a subset of patients with pain and multiple concurrent risk factors, such as persistent poorly controlled diabetes and either reduced blink or a history of corneal procedures, the panel strongly recommended testing given the possibility of neuropathic corneal pain or pain from generalized inflammation.

In patients in whom the index of suspicion is lower, the panel suggested considering (median rating 4–6) corneal sensitivity testing but did not strongly recommend it. These scenarios include most patients with newly observed corneal epithelial dye staining and persistent poorly controlled diabetes, and patients with acquired limbal stem cell deficiency. NK is generally not diagnosed in the absence of epithelial disease; however, in order to detect early loss of sensation, the panel agreed that corneal sensitivity testing may be considered in patients with persistent poorly controlled diabetes and vision changes not ascribed to diabetic retinopathy or cataract, even in the absence of corneal findings.

### Tests for diagnosis and staging

The panel agreed that the optimal way to diagnose and stage NK was to perform a test of corneal nerve function (either quantitative or qualitative), in conjunction with a slit lamp examination with vital staining and measurement of any observed epithelial defect.

### Treatments

Multiple treatments may be used concurrently, particularly in more advanced disease. As a result of differences in insurance coverage, technical expertise, or other reasons affecting access to care, not all treatments recommended by the panel will be available in all circumstances. Table 3 summarizes the treatments rated as optimal (median 7–9) in at least one NK disease stage. In addition to those listed in the table, the panel agreed optimal care required identifying and treating underlying causes of NK in all patients. These treatments may include antiviral medication in the case of herpetic disease, correcting eyelid abnormalities and treating meibomian gland dysfunction, anti-inflammatory medications for stromal inflammation or neovascularization, supportive therapy for limbal stem cell deficiency, and controlling hemoglobin A1c levels in patients with diabetes. In addition, the panel agreed patients with NK should be evaluated for ocular side effects of systemic therapies such as neuroleptic, antipsychotic, oncology, and antihistamine drugs.

**Table 3:** Potential treatments by neurotrophic keratopathy stage (rated as optimal in at least one stage)

	Early/less severe (e.g., Stage 1)	Moderate (e.g., Stage 2)	Later/more severe (e.g., Stage 3)
Discontinue all preservative-containing topical medications	^a^	^a^	^a^
**Medical management**			
Topical preservative-free drops	^a^	^a^	^a^
Topical preservative-free gel drops or ointments	^a^	^a^	^a^
Autologous serum drops, human umbilical cord serum, platelet rich plasma	^a^	^a^	^a^
Recombinant human nerve growth factor (cenegermin)	^b^	^a^	^a^
Prophylactic topical preservative-free antibiotics (excluding aminoglycosides)		^a^	^a^
Matrix metalloproteinases inhibitors	^b^	^a^	^a^
**Non-surgical intervention (i.e., office procedures)**			
Corneal therapeutic contact lenses	^b^	^a^	^b^
Fresh-frozen self-retained amniotic membrane		^a^	^a^
Punctal occlusion	^a^	^a^	^a^
Synthetic (cyanoacrylate) tissue adhesive			^a^
**Surgical intervention (i.e., operating room procedures)**			
Tarsorrhaphy		^b^	^a^
Amniotic membrane transplant		^b^	^a^
Corneal neurotization		^b^	^a^

^a^Treatments rated as potentially optimal, depending on the patient’s individual circumstances

^b^Treatments rated as potentially appropriate, depending on the patient’s individual circumstances (not noted in the manuscript)

For all stages of NK, the panel agreed that optimal care included discontinuing preservative-containing topical medications to the extent possible, recognizing that some topical medications do not have preservative-free alternatives (in which case the panel recommended working to decrease the dose). The panel concluded that for all patients with NK (regardless of stage), optimal treatments (alone or in combination, depending on the circumstances) may include: preservative-free tear substitution or lubricants (including gels and ointments), punctal occlusion, and autologous serum tears/umbilical cord serum drops/platelet rich plasma drops. For patients with Stage 2 disease, the panel considered cenegermin, prophylactic topical preservative-free antibiotics, matrix metalloproteinases inhibitors such as oral tetracyclines (e.g., doxycycline), corneal therapeutic contact lenses, and fresh-frozen self-retained amniotic membrane to be additional optimal treatments. For Stage 3 disease, in addition to the treatments recommended in Stage 2, the panel agreed that synthetic tissue adhesive, tarsorrhaphy, amniotic membrane transplant, and corneal neurotization were optimal treatments.

## Discussion

In the current study, expert clinicians reviewed published evidence and independently rated 735 patient scenarios to arrive at consensus recommendations on when to screen for and how best to diagnose and treat NK. Diagnosis may be delayed in NK because patients experience few symptoms [1], however, the best opportunity to reverse ocular surface damage and prevent progression is early in the disease course [6]. While reviews on how to diagnose and treat NK have been published [1, 2, 7], none used a methodologically rigorous process.

The current study was not a clinical study of patient care, but rather used detailed scenarios to help clinicians identify and articulate best practice. The resulting consensus statements align with existing evidence. The panel’s guidance for when to conduct a test of corneal sensitivity to screen for NK aligns with known etiologies of NK. For example, retrospective analyses of patients with NK have found common causes of NK or conditions associated with NK to be a history of herpetic eye disease [1, 7, 25, 26], post-surgical or condition-caused nerve damage [7], syndromes that may reduce blink such as Bell’s palsy or Parkinson’s disease [27], and repeated corneal procedures [25]. While studies have not specifically estimated the prevalence of NK in patients with diabetes, several studies report diabetes to be a common cause of NK [25] and suggested that progression in diabetes, including diabetic neuropathy and end-organ damage, are associated with the precursors to NK [28–35]. Further, in a large, retrospective matched cohort study, patients with diabetes were 1.31 times more likely to develop corneal ulceration than matched controls [36].

Treatments included in the panel’s recommendations have been shown to be effective in treating NK. For example, autologous serum eye drops have been shown to be effective in retrospective, noncomparative case series [37] and there is evidence that human umbilical cord serum and platelet rich plasma drops can be used to treat ocular surface conditions [38–40]. Cenegermin is currently the only US Food and Drug Administration approved treatment for NK. Its efficacy and safety have been demonstrated in patients with Stage 2–3 NK in two Phase II trials [19, 20] and one case series [41]. Matrix metalloproteinases inhibitors were shown to be effective in several small studies [42] of patients with NK. Successful outcomes were noted in two studies reporting on the use of self-retained amniotic membrane in patients with NK [43, 44] and sutured or glued amniotic membrane transplant has been shown to be effective for healing NK ulcers [21]. In two studies of NK patients treated with corneal neurotization, patients demonstrated improvements in pain, NK stage, visual acuity, and corneal sensation [45, 46]. There are also at least five ongoing clinical trials of treatments for patients with NK [47].

To develop these statements, we used the RAND/UCLA modified Delphi panel method which has been used extensively to develop quality measures and clinical guidance in a variety of areas [8]. There is published evidence that guidelines developed using this method have content, construct, and predictive validity [9]. The method has been shown to produce guidance that improves health outcomes [10–12]. Ratings of appropriateness from this method have been found to be reliable with test-retest reliability > 0.9 using the same panelists 6–8 months later [48] and kappa statistics across several panels with different members similar to those of some common diagnostic tests [49]. Independent panels using this method also produce similar ratings to one another, although the degree of similarity depends on the level of evidence available. A review of Delphi panels showed 90% agreement among the panels that used randomized control trial evidence compared to 70–80% agreement in the panels which used a weaker evidence base [49].

Our study has several limitations. First, this study is descriptive only and the relationship between our screening, diagnosis, and treatment recommendations and patient outcomes has yet to be demonstrated. No patient data was collected to develop our recommendations nor used to test their validity. Second, there are few large studies on the identification or treatment of NK, so these consensus statements reflect studies with small sample sizes, observational studies, and the experience of a single group of clinicians. Nevertheless, we used the RAND/UCLA modified Delphi panel method to develop these statements, which as noted above, has been shown to be reliable. Third, our broad recommendations likely do not capture nuances encountered in real-world practice or individual patient circumstances. Further, scenarios were developed collaboratively with experts and rated by experts, which may have resulted in simpler scenarios that excluded characteristics that could have generated more debate. We recognize that in practice, clinicians will have to consider many other clinical and non-clinical factors beyond those addressed in our statements. Fourth, while we chose to focus our guidelines on how to treat NK rather than the underlying cause of NK, we acknowledge that treating the disease etiology and concurrent inflammation are also essential to patient care [1]. Lastly, our panel consisted of experts from the US only, so our guidelines may not be generalizable to other countries.

## Conclusion

The guidance described here reflects agreement among a panel of experts using a methodologically sound process on when to screen for and how best to diagnose and treat NK based on currently available evidence. We believe this guidance could improve the quality of care for NK patients by helping to diagnose patients earlier in their disease course when progression can be reduced or stopped and by recommending evidence-based treatments for each disease stage. Studies to demonstrate whether our recommendations improve health outcomes will further advance the management of this relatively rare but serious disease.

## Data Availability

Data sharing is not applicable to this article as no datasets were generated or analyzed during the current study.

## Abbreviations

NK: Neurotrophic keratopathy
US: United States
